# Hydrophobic analogues of rhodamine B and rhodamine 101: potent fluorescent probes of mitochondria in living *C. elegans*

**DOI:** 10.3762/bjoc.8.243

**Published:** 2012-12-11

**Authors:** Laurie F Mottram, Safiyyah Forbes, Brian D Ackley, Blake R Peterson

**Affiliations:** 1 Department of Medicinal Chemistry, The University of Kansas, Lawrence, KS 66045, United States; 2 Department of Molecular Biosciences, The University of Kansas, Lawrence, KS 66045, United States

**Keywords:** Caenorhabditis elegans, chemical biology, fission, fluorophores, fluorescence, fusion, imaging, in vivo, microscopy, mitochondria, model organisms, organelle, rhodamine, spectroscopy

## Abstract

Mitochondria undergo dynamic fusion and fission events that affect the structure and function of these critical energy-producing cellular organelles. Defects in these dynamic processes have been implicated in a wide range of human diseases including ischemia, neurodegeneration, metabolic disease, and cancer. To provide new tools for imaging of mitochondria in vivo, we synthesized novel hydrophobic analogues of the red fluorescent dyes rhodamine B and rhodamine 101 that replace the carboxylate with a methyl group. Compared to the parent compounds, methyl analogues termed HRB and HR101 exhibit slightly red-shifted absorbance and emission spectra (5–9 nm), modest reductions in molar extinction coefficent and quantum yield, and enhanced partitioning into octanol compared with aqueous buffer of 10-fold or more. Comparison of living *C. elegans* (nematode roundworm) animals treated with the classic fluorescent mitochondrial stains rhodamine 123, rhodamine 6G, and rhodamine B, as well as the structurally related fluorophores rhodamine 101, and basic violet 11, revealed that HRB and HR101 are the most potent mitochondrial probes, enabling imaging of mitochondrial motility, fusion, and fission in the germline and other tissues by confocal laser scanning microscopy after treatment for 2 h at concentrations as low as 100 picomolar. Because transgenes are poorly expressed in the germline of these animals, these small molecules represent superior tools for labeling dynamic mitochondria in this tissue compared with the expression of mitochondria-targeted fluorescent proteins. The high bioavailabilty of these novel fluorescent probes may facilitate the identification of agents and factors that affect diverse aspects of mitochondrial biology in vivo.

## Introduction

**Figure 1:**
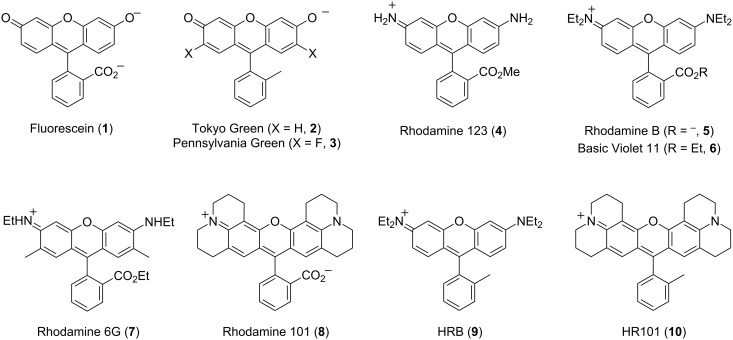
Structures of known fluorophores (**1**–**8**) and novel hydrophobic analogues of rhodamine (HRB, **9**, and HR101, **10**).

Fluorescent molecular probes represent critical tools for studies of chemical biology [1]. These compounds allow the creation of sensitive enzyme substrates, sensors of a wide variety of analytes, and specific markers of cellular organelles and other components. Although many structurally diverse fluorophores have been reported, many common fluorophores such as dianionic fluorescein (**1**, Figure 1) are defined by highly polar conjugated π systems. This high polarity confers substantial aqueous solubility, which is beneficial for some applications, such as protein labeling, but also results in low cellular permeability in assays involving living cells. Hydrophobic analogues of fluorescein such as Tokyo Green (**2**) [2], Pennsylvania Green (**3**) [3–4], and others [5] have been synthesized that replace a carboxylate with a methyl group or other less polar functionality. These hydrophobic analogues are generally more effective at penetrating cellular membranes [2,4].

Rhodamines such as rhodamine 123 (**4**), rhodamine B (**5**), basic violet 11 (**6**), rhodamine 6G (**7**), and rhodamine 101 (**8**) are renowned for their red-shifted fluorescence, photostability, and high quantum yields over a wide range of pH values (i.e., pH 4–10). These fluorophores penetrate cells more readily than analogous fluorescein derivatives, because the negative plasma membrane potential within the cytoplasm of cells (typically −30 to −60 mV) represents less of a barrier to entry to cations and zwitterions, compared with anionic compounds. Moreover, the delocalized cationic π-system of rhodamines further promotes their accumulation in energy-producing mitochondria, due to the even stronger negative potential across mitochondrial inner membranes (typically −120 to −180 mV, depending on cell type) [6–9]. This membrane potential is critical for ATP synthesis, and is generated by pumping of protons across the mitochondrial inner membrane by the respiratory chain. Many delocalized lipophilic cations preferentially accumulate in these organelles, and some rosamines, i.e., compounds that lack the carboxylate of rhodamines [10], have been shown to potently depolarize mitochondria. Elevated mitochondrial membrane potential is a hallmark of cancer cell lines [11–12], and depolarization of mitochondria by rhodamines and rosamines can confer selective anticancer activity both in vitro and in vivo in animal models [13].

Under nontoxic conditions, rhodamines and mitochondria-targeted fluorescent proteins have been extensively used for imaging and analysis of these organelles in cell culture, enabling studies of dynamic fusion and fission events that are critical for mitochondrial structure and function [8,14–17]. Agents and factors that affect fusion and fission of mitochondria are of substantial interest [18] because disregulation of these dynamic processes has been implicated in a wide range of human diseases, including ischemia, neurodegeneration, metabolic disease, and cancer [19–20].

In vivo, the pharmacokinetics of some rhodamines have been evaluated [21–23], and some have been used for imaging of mitochondria in the optically transparent model organism *C. elegans* (nematode worm) [24–25]. However, rhodamine 123 (**4**) [25], rhodamine B (**5**) [26], rhodamine 6G (**7**) [24], rhodamine B hexyl ester [27], and tetramethylrhodamine ethyl ester [27] are of low potency in living *C. elegans*, typically requiring treatment times of up to 48 h [24], often at concentrations as high as 30 µM [27]. This low potency may have led some investigators interested in imaging fusion and fission of mitochondria in *C. elegans* [28] to forego the use of small-molecule fluorescent probes and instead to use time-consuming molecular biology methods to generate transgenic animals that express fluorescent proteins, such as mitoGFP, that are targeted to this organelle. In general, the choice to use small-molecule probes or molecular-biology-based approaches for these types of imaging applications can be challenging because of our limited understanding of the bioavailability and bioaccumulation of small molecules in this model organism [29–34]. In general, these soil-dwelling nematodes are considered to be substantially less pemeant to small molecules than other animals, and most drug-like compounds do not efficiently accumulate in worms [30]. Consequently, to observe biological effects, many pharmacological agents must be added to *C. elegans* at concentrations orders of magnitude higher than are used with mammalian cells in culture [35–36]. For some hydrophobic compounds, delivery systems [31–32] can improve their uptake.

We hypothesized that poor bioavailability of rhodamines in *C. elegans* may be responsible for their low potency as probes of mitochondria in this organism. We reasoned that the relatively high polarity of these charged compounds (e.g., LogD rhodamine 123 (**4**) = 0.53 [37], −0.62 [9]), offering functional groups for possible xenobiotic metabolism in the intestine or other tissues [29–30], may limit absorption. Rhodamine esters such as **4**, **6**, and **7** may also be substrates of esterases [38] in vivo, resulting in the production of more polar fluorophores that may be inefficiently absorbed. To test this hypothesis, we synthesized novel hydrophobic analogues of rhodamine B (**5**) and rhodamine 101 (**8**) that replace the carboxylate with a methyl group (Figure 1). The resulting analogues, termed HRB **9** and HR101 **10**, allowed evaluation of how subtle changes in chemical structure impact photophysical and physicochemical properties and the utility of rhodamines and analogues for imaging mitochondria in *C. elegans*. These studies revealed that the hydrophobic rosamines HRB **9** and HR101 **10** represent highly potent and selective fluorescent probes of these organelles. Treatment of *C. elegans* with these compounds for as little as two hours at concentrations as low as 100 pM enables selective imaging of mitochondria in vivo, including visualization of the dynamics of fusion and fission of these organelles in the germline of living animals.

## Results and Discussion

### Synthesis of fluorophores

**Scheme 1:**
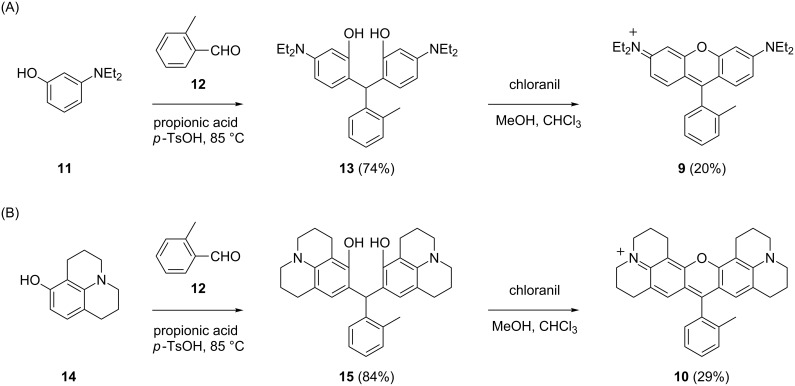
Synthesis of the HRB **9** and HR101 **10** fluorophores.

As shown in Scheme 1, the triarylmethane scaffolds of HRB **9** and HR101 **10** were synthesized by condensation of the corresponding dialkyaminophenol with *o*-tolualdehyde (**12**) [39]. Oxidative cyclization of triarylmethanes with the quinone oxidant chloranil provided **9** and **10** in modest yield. 8-Hydroxyjulolidine (**14**) for synthesis of HR101 **10** was either purchased commercially or prepared as previously described [40].

### Photophysical and physicochemical properties

**Figure 2:**
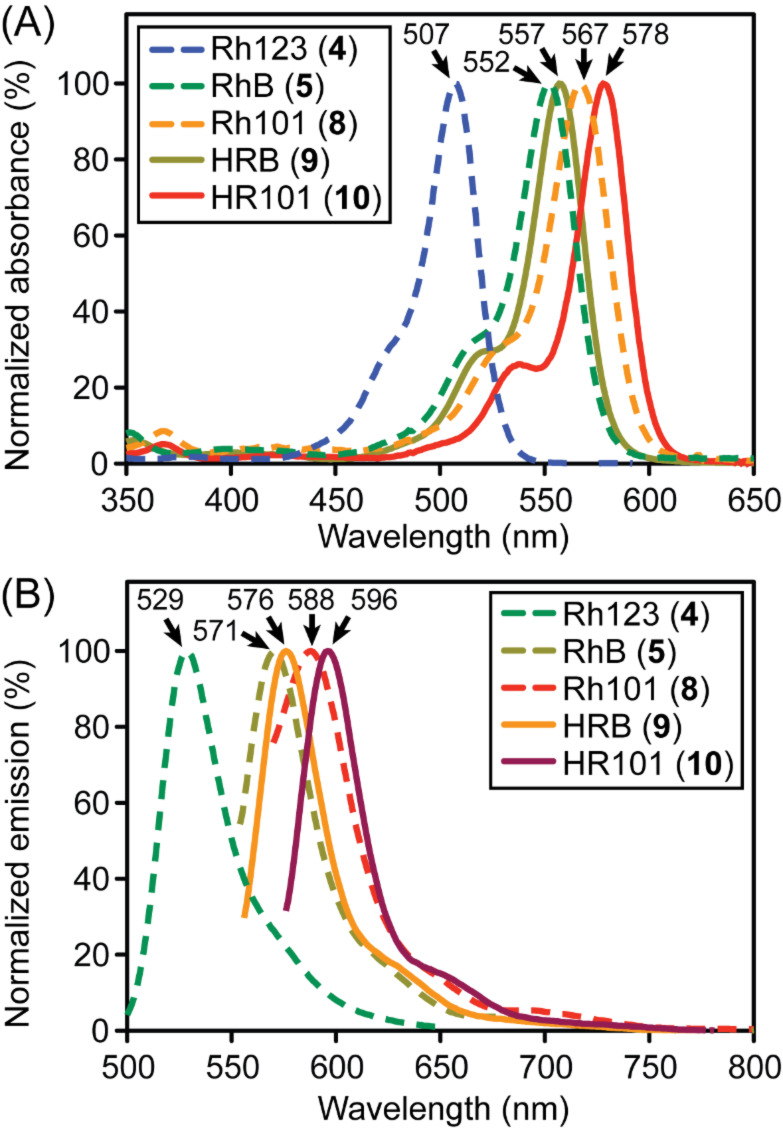
Normalized absorbance (Panel A) and fluorescence emission (Panel B) spectra. Fluorophores were analyzed at 10 µM (absorbance) or 5 nM (fluorescence) in MeOH. The spectra for rhodamine 123 (**4**) in MeOH were downloaded from a publically accessible database [41]. Maximum absorbance and emission wavelengths are indicated with arrows.

The absorbance (panel A) and fluorescence emission (panel B) spectra of fluorophores **4**, **5**, and **8**–**10** are shown in Figure 2.

**Figure 3:**
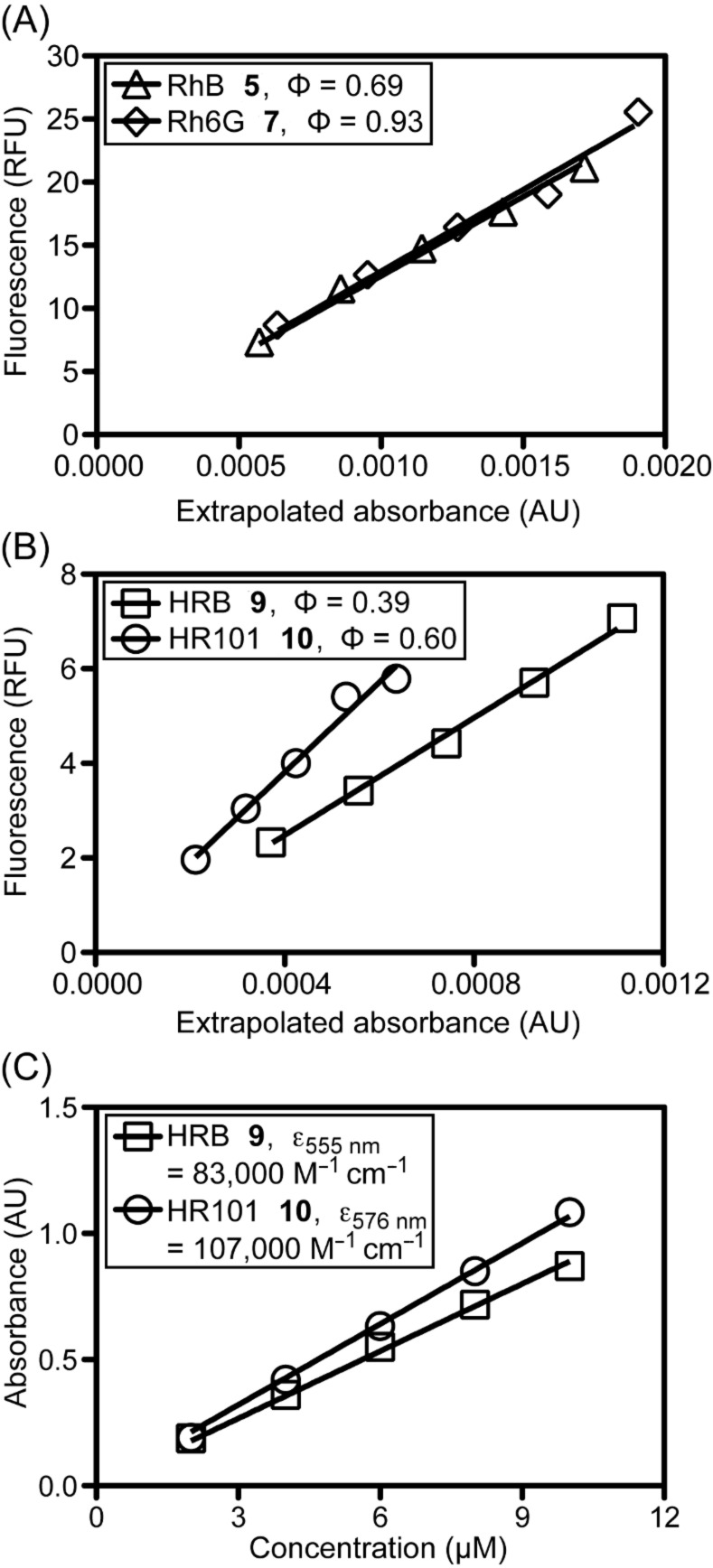
Linear regression used to determine spectroscopic parameters of HRB **9** and HR101 **10** in MeOH. Panels A and B: Determination of quantum yields relative to rhodamine B (**5**) [44] and rhodamine 6G (**7**) [45]. Panel C: Quantification of molar extinction coefficients.

The red-shifted spectra of HR101 **10** compared with HRB **9** can be explained by greater delocalization of the lone pairs on nitrogen due to restricted rotation about C–N bonds conferred by the fused rings of **10**. By using the method of Williams [42], the quantum yields of **9** and **10** were determined relative to rhodamine B (**5**) and rhodamine 6G (**7**, Figure 3, panels A and B), and the extinction coefficients of these compounds were quantified (Figure 3, panel C). The replacement of the carboxylate of rhodamine B (**5**) and rhodamine 101 (**8**) with a methyl group slightly red shifted the absorbance and emission spectra in methanol (5 nm and 8 to 9 nm, respectively). This change also decreased the molar extinction coefficient, as compared with the reported [43] molar extinction coefficient of rhodamine B (**5**) of 106,000 M^−1^ cm^−1^ (at 545 nm in ethanol) with HRB (**9**, 83,000 M^−1^ cm^−1^ at 555 nm in methanol). Additionally, a modest reduction in quantum yield of ~0.3 for both fluorophores was observed.

Spectroscopic studies revealed that HRB **9** and HR101 **10** are not as bright as the carboxylate-containing parent dyes. However, because the fluorescence intensity is directly proportional to the product of the extinction coefficient and the quantum yield at excitation levels below saturation, these analogues represent very bright fluorophores for imaging applications. Potentially more important for activity in vivo, replacement of the carboxylate of rhodamine B (**5**) and rhodamine 101 (**8**) with a methyl group was predicted to substantially enhance hydrophobicity. Previously reported measurements of log *D* in octanol/neutral buffer solutions of rhodamine 123 (**4**), rhodamine B (**5**), and rhodamine 6G (**7**), measurements of the log *D* of rhodamine 101 (**8**), HRB **9**, and HR101 **10** by using a fluorescence-based shake-flask method, and *c*·log *P* values for compounds **4**–**10** calculated by using a recent version of CambridgeSoft ChemBioDraw software, are shown in Table 1. Although some differences exist between the calculated and measured values, the relative trends illustrate how structural modifications of these compounds are likely to affect fluorophore solubility and cellular permeability.

**Table 1:** Partition constants of fluorophores.

Fluorophore	c·log *P*^a^	log *D*_octanol/buffer_

Rhodamine 123 (**4**)	1.5	0.5 [37], −0.6 [9]
Rhodamine B (**5**)	−1.1	2.3 [46]
Basic Violet 11 (**6**)	3.7	N.D.^b^
Rhodamine 6G (**7**)	6.5	2.1 [37]
Rhodamine 101 (**8**)	3.8	5.2^c^
HRB **9**	3.4	5.9^c^
HR101 **10**	8.6	6.2^c^

^a^Calculated with ChemBioDraw Ultra, version 12.0.3, from structures bearing functional groups at ionization states predicted to dominate at pH 7 (as shown in Figure 1). ^b^N.D. Not determined. ^c^Determined in octanol/buffer_pH 7.4_ by using a fluorescence-based shake-flask method.

### Imaging of fluorophores in vivo by confocal microscopy

**Figure 4:**
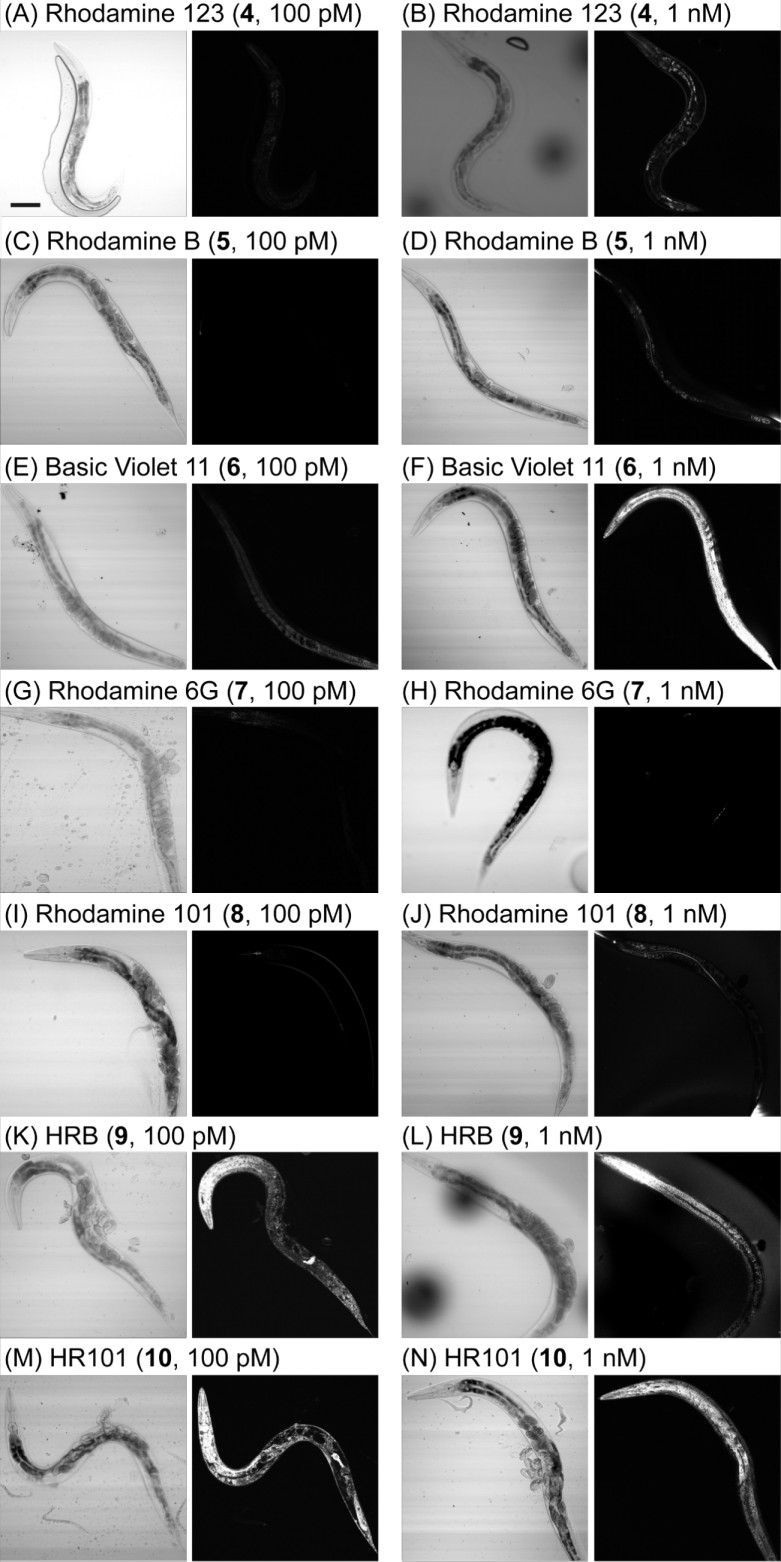
Differential interference contrast (DIC, left panels) and confocal laser scanning (right panels) micrographs of living *C. elegans* (20× objective) treated with synthetic compounds for 2 h followed by transfer to an imaging pad containing polystyrene beads for immobilization. The weak signal observed with rhodamine 123 at 1 nM (panel B) was independently identified as autofluorescence of the intestine. Animals are oriented with the anterior end (head) to the left and the ventral side (base) down and/or toward the left. Scale bar = 100 microns.

To visualize the absorption and distribution of synthetic rhodamines and analogues in vivo, living adult *C. elegans* were initially subjected to an acute treatment followed by confocal laser scanning microscopy of mechanically immobilized whole animals (20× objective). In contrast to previous reports where treatment with rhodamines for 36–48 h was required [24,26], treatment with HRB **9** and HR101 **10** yielded observable fluorescence in some animals within 30 min, with most animals becoming fluorescent within 2 h. In the assay shown in Figure 4, adult animals were treated with rhodamine 123 (**4**), rhodamine B (**5**), basic violet 11 (**6**), rhodamine 6G (**7**), rhodamine 101 (**8**), HRB (**9**), and HR101 (**10**) at identical fixed concentrations of 100 pM or 1 nM for 2 h. Among the fluorophores examined, HRB **9**, HR101 **10**, and basic violet 11 (**6**) showed the highest bioaccumulation, and the fluorescence of these three compounds could be detected at much lower concentrations than the more polar rhodamine 123 (**4**), rhodamine B (**5**), rhodamine 6G (**7**), and rhodamine 101 (**8**) fluorophores. Although basic violet 11 (**6**) was relatively potent, this compound appeared to be of low selectivity, staining multiple intracellular structures including membranes, mitochondria and nuclei, and occasionally was observed in the cytosol of cells of living animals. Unlike all of the other fluorophores tested, HRB **9** and HR101 **10** engendered strong fluorescence in vivo after 2 h at concentrations as low as 100 pM.

**Figure 5:**
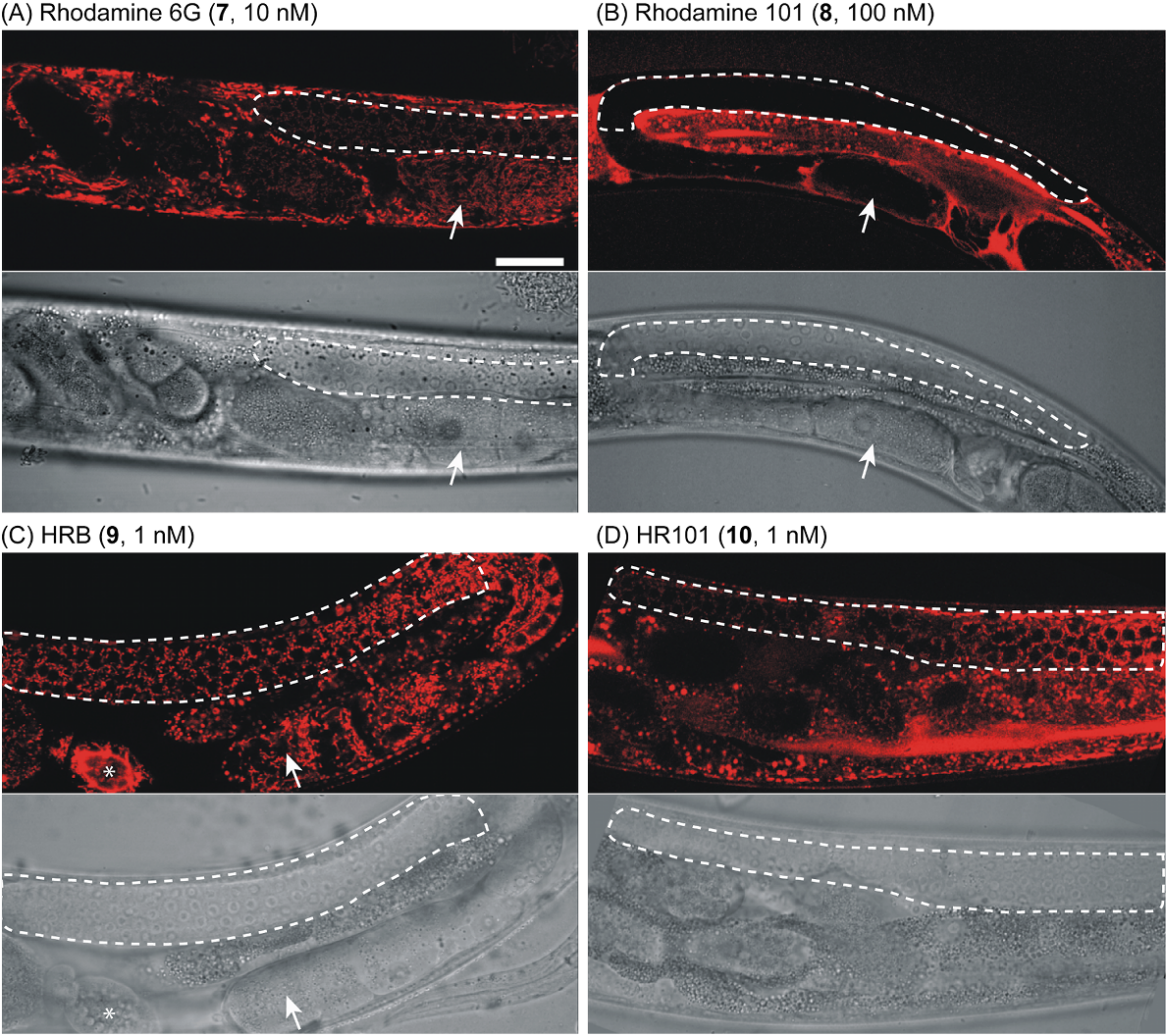
Panels A–D: High magnification confocal (top panels) and DIC micrographs (bottom panels, 60× objective) of young adult *C. elegans* after treatment with fluorophores for 2 h. The distal gonad containing the germline is shown marked by white dashed lines, the anterior of the animal is to the left, and ventral is down. In Panels C, and D, strong labeling of tubular mitochondria surrounding the mitotic nuclei of the gonad can be observed as well as strong staining of mitochondria within oocytes (white arrows indicate the most proximal oocyte, where visible) and the fibrous organelle (marked with an asterisk in panel C) of spermatheca. Scale bar = 25 microns.

**Figure 6:**
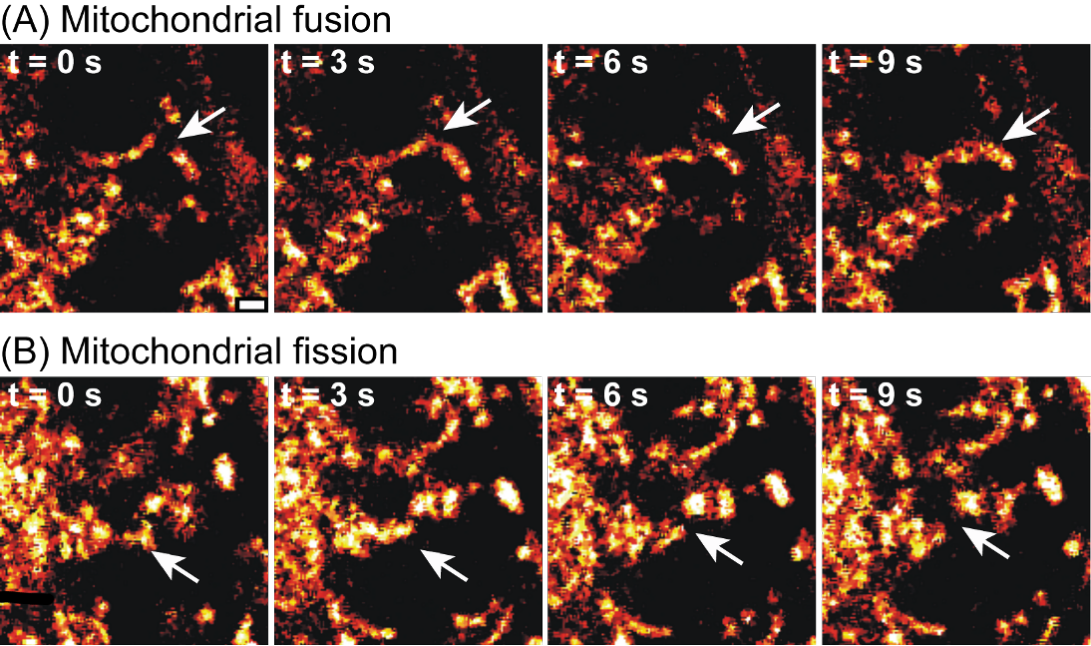
Images of mitochondrial motility, fusion, and fission in the germline of *C. elegans* extracted from confocal video microscopy. White arrows indicate fusion and fission of specific mitochondria. Prior to imaging, animals were treated with HRB (**9**, 1 nM, 2 h). Scale bar = 1 micron.

Higher magnification confocal microscopy (60× objective) using a variety of fluorophore concentrations revealed that most cells of living *C. elegans* accumulate the fluorophores rhodamine 6G (**7**), HRB **9**, and HR101 **10** after treatment for 2 h (Figure 5). Under these conditions, the more polar rhodamine 101 (**8**) was observed to be excluded from some cells and tissues. Labeling of specific organelles by rhodamine 6G (**7**), HRB **9**, and HR101 **10** was observed in hypodermis, muscle, neurons (data not shown), and the germline of these animals (Figure 5). Examination of the pattern of staining in the germline, part of the distal gonad containing a population of germ cells that lack complete borders, demonstrated that the more specific rhodamine derivatives illuminate tubular or punctate organelles (Figure 5 and Figure 6) that are highly motile (Figure 6 and movie, Supporting Information File 1), consistent with predominant accumulation in mitochondria. These structures elongated and contracted (see movie, Supporting Information File 1) and underwent fusion and fission (Figure 6) consistent with other studies of mitochondria in *C. elegans* [24]*.* Comparative imaging indicated that rhodamine 6G (**7**), HRB **9**, and HR101 **10** represent the most specific mitochondrial stains, with similar profiles of mitochondrial labeling within the germline, but the more hydrophobic fluorophores exhibited 10-fold higher potency. In contrast, the more polar rhodamine 101 (**8**), even at a 100-fold higher concentration compared with HRB **9** and HR101 **10**, did not penetrate into the germline under these conditions (Figure 5).

## Conclusion

Genetically encoded fluorophores such as green fluorescent protein (GFP) have revolutionized cell biology and studies of physiological processes. However, fluorescent small-molecule probes continue to offer advantages for some imaging applications. One advantage illustrated here is the ability to examine mitochondrial fusion and fission dynamics in the germline of living *C. elegans* after acute treatment with the hydrophobic rhodamine analogues HRB **9** and HR101 **10**. In this tissue, genetically encoded proteins are unsuitable for imaging of mitochondria, because transgenes are poorly expressed in the germline of *C. elegans*. However, genetically encoded fluorescent markers are advantageous in other contexts in that they can be more easily spatially confined to specific tissues of *C. elegans* by using cell-type specific promoters to drive gene expression. The rapid accumulation (within 2 h) and high potency (effective at ≥ 100 pM) of HRB **9** and HR101 **10** can be contrasted with previous studies [26] of rhodamine B (**5**) that employed treatment with 2 µM for 36 h to image mitochondria in the germline of *C. elegans.* These new probes rapidly accumulate in mitochondria at low concentrations because of their more favorable pharmacokinetic properties.

Animals treated with HRB **9** and HR101 **10** at 10 nM for 2 h were viable and exhibited grossly wild-type movement, suggesting that mitochondrial function remains largely if not completely intact during labeling with low concentrations of these probes. In contrast, treatment with much higher concentrations of these probes (e.g., ≥1 mM) under these conditions conferred some toxicity (data not shown). By comparison, treatment of *C. elegans* with the mitochondrial poison sodium azide at 25–50 mM rapidly causes paralysis and eventually causes death. Consequently, the high potency, low apparent toxicity, and rapid uptake of HRB **9** and HR101 **10** by *C. elegans* has the potential to be useful in a variety of applications. For screening purposes, these compounds may be used to rapidly examine multiple genotypes of interest without the need to introduce the genetically encoded sensor to each genetic background under investigation. Alternatively, a genetically encoded fluorescent protein may be complemented with these molecular probes to perform experiments such as fluorescence recovery after photobleaching (FRAP), where one of the markers remains unbleached. Chemical probes also have a variety of half-lives in vivo, a property that may be beneficial in some assays compared to long-lived fluorescent markers, such as mitoGFP and related proteins. High-content screening of chemical libraries against *C. elegans* treated with HRB **9** and HR101 **10** may facilitate the identification of inhibitors of mitochondrial fusion or fission effective in vivo, potentially enabling the discovery of new leads for the treatment of diseases associated with dysfunctional mitochondria.

A challenge associated with chemical biology studies in *C. elegans* is the low permeability of this animal to small molecules. For example, many bioactive compounds require at least a 10-fold higher dose to exhibit activity in *C. elegans* compared to other organisms [30], due to a number of physical and enzymatic barriers to entry of small molecules. On the exterior surface of *C. elegans*, the cuticular exoskeleton of these animals is a highly cross-linked carbohydrate-rich outer layer that limits access of molecules to the epidermis for potential uptake. For oral administration, molecules that are ingested by these animals must pass through an intestine replete with a wide range of xenobiotic detoxification enzymes, including cytochrome P450s, phase-II transferases, and efflux pumps such as P-glycoprotein [35], to reach target tissues [29]. Thus, compounds such as HRB **9** and HR101 **10** that show high potency in this animal have passed through a stringent biological filter, providing a basis to justify studies in more complex and costly animal models. Fluorescence-imaging studies of small molecules in *C. elegans* have the potential to provide a deeper understanding of molecular modifications that facilitate the access of compounds to targets in vivo and may improve our ability to design more effective therapeutics and probes.

## Experimental

### Synthesis

Chemical reagents were obtained from Acros, Aldrich, Alfa Aesar, or TCI America. Solvents were from EM Science. Commercial grade reagents were used without further purification unless otherwise noted. Anhydrous solvents were obtained after passage through a drying column of a solvent-purification system from GlassContour (Laguna Beach, CA). All reactions were performed under an atmosphere of dry argon or nitrogen. Reactions were monitored by analytical thin-layer chromatography on plates coated with 0.25 mm silica gel 60 F_254_ (EM Science). TLC plates were visualized by UV irradiation (254 nm) or stained with a solution of phosphomolybdic acid and sulfuric acid in ethanol (1:1:20). Flash column chromatography employed ICN SiliTech Silica Gel (32–63 μm). Melting points were measured with a Thomas Hoover capillary melting point apparatus and are uncorrected. Infrared spectra were obtained with a Perkin Elmer 1600 Series FTIR. NMR spectra were obtained with Bruker CDPX-300, DPX-300, or DRX-400 instruments with chemical shifts reported in parts per million (ppm, δ) referenced to either CDCl_3_ (^1^H, 7.27 ppm; ^13^C, 77.23 ppm), or DMSO-*d*_6_ (^1^H, 2.50 ppm; ^13^C, 39.51 ppm). High-resolution mass spectra were obtained from mass spectrometry facilities at the University of Kansas or The Pennsylvania State University (ESI and TOF). Peaks are reported as *m*/*z*. Absorbance spectra were obtained with an Agilient 8453 UV–vis spectrophotometer. Fluorescence measurements employed either a PTi MD-5020 or a Perkin-Elmer LS55 fluorescence spectrometer with a 10 nm excitation slit width.

**5-(Diethylamino)-2-{[4-(diethylamino)-2-hydroxyphenyl](2-methylphenyl)methyl}phenol (13):** 3-Diethylaminophenol (**11**, 500 mg, 3.0 mmol) and *o*-tolualdehyde (**12**, 175 µL, 1.5 mmol) were dissolved in propionic acid (15 mL) and catalytic *p*-TsOH acid was added. The reaction was purged with argon and heated to 80–85 °C. The reaction was monitored by TLC by quenching aliquots (100 µL) in aqueous saturated sodium bicarbonate (200 µL), followed by extraction of the organic material with EtOAc (200 µL). When all starting materials had been consumed (5 h), the solution was cooled to 0 °C and poured into excess aqueous sodium acetate (3 M, 100 mL) to neutralize the propionic acid and precipitate the triarylmethane. The filtrate was collected by vacuum filtration, washed with copious amounts of water, and dried under reduced pressure to provide **13** (480 mg, 74%) as a tan solid. Mp 68–70 °CM; ^1^H NMR (400 MHz, CDCl_3_) δ 7.02 (m, 3H), 6.90 (d, *J* = 6.8 Hz, 1H), 6.51 (d, *J* = 8.3 Hz, 1H), 6.05 (m, 4H), 5.55 (s, 1H), 3.10 (q, *J* = 6.9 Hz, 8H), 2.10 (s, 3H), 0.98 (t, *J* = 6.9 Hz, 12H); ^13^C NMR (75 MHz, CDCl_3_) δ 154.8, 148.1, 140.8, 136.8, 130.4, 130.4, 130.2, 115.2, 105.0, 104.8, 100.2, 100.0, 44.2, 40.8, 19.4, 12.4; IR (film) ν_max_: 3457, 2971, 1620, 1521 cm^−1^; HRMS (ESI^+^, TOF, *m*/*z*): [M + H]^+^ calcd for C_28_H_36_O_2_N_2_, 433.2850; found, 433.2848.

**6-(Diethylamino)-*N*,*N*-diethyl-9-(2-methylphenyl)-3*H*-xanthen-3-imine (HRB, 9):** 5-(Diethylamino)-2-{[4-(diethylamino)-2-hydroxyphenyl](2-methylphenyl)methyl}phenol (**13**, 100 mg, 0.23 mmol) and chloranil (85 mg, 0.34 mmol) were dissolved in MeOH/CHCl_3_ (1:1, 10 mL). The solution was stirred at 23 °C for 1 h. When the reaction was complete as evidenced by TLC, the solvent was removed under reduced pressure and the residue was applied directly to a column of silica gel. Flash chromatography (MeOH/CHCl_3_ 1:9) afforded **9** (21 mg, 20%) as a purple film of the chloride salt. ^1^H NMR (300 MHz, CDCl_3_) δ 7.54–7.38 (m, 3H), 7.16–7.13 (m, 3H), 6.86–6.79 (m, 4H), 3.62 (q, *J* = 7.2 Hz, 8H), 2.06 (s, 3H), 1.33 (t, *J* = 7.1 Hz, 12H); ^13^C NMR (75 MHz, CDCl_3_) δ 157.9, 157.8, 155.6, 135.8, 131.6, 131.4, 130.7, 130.1, 128.7, 126.1, 114.0, 113.4, 96.5, 46.0, 19.5, 12.4; IR (film) ν_max_: 2981, 1590, 1179 cm^−1^; HRMS (ESI^+^, TOF, *m*/*z*): [M]^+^ calcd for C_28_H_33_ON_2_, 413.2587; found, 413.2583.

**9,9'-[(2-Methylphenyl)methylene]bis(2,3,6,7-tetrahydro-1*H*,5*H*-pyrido[3,2,1-*ij*]quinolin-8-ol) (15):** 8-Hydroxyjulolidine (**14**, 500 mg, 2.6 mmol) and *o*-tolualdehyde (**12**, 154 µL, 1.3 mmol) were dissolved in propionic acid (15 mL) and catalytic *p*-TsOH acid was added. The reaction was performed as described for the preparation of **13** to afford **15** (525 mg, 84%) as a gray solid. Mp 170–174 °C; ^1^H NMR (400 MHz, CDCl_3_) δ 7.12–7.04 (m, 3H), 6.73 (d, *J*_1_* =* 7.5 Hz, 1H), 6.34 (3, 2H), 6.02 (s, 1H), 3.35 (m, 4H), 3.24 (m, 4H), 2.69 (m, 8H), 2.12 (s, 3H), 2.10 (m, 8H); ^13^C NMR (75 MHz, CDCl_3_) δ 150.3, 139.8, 136.1, 132.5, 129.8, 127.9, 127.7, 125.8, 125.0, 119.6, 116.6, 52.1, 51.5, 39.9, 24.8, 20.1, 19.7, 19.1, 18.5 (× 2); IR (film) ν_max_: 3270, 2953, 1673, 1471 cm^−1^; HRMS (ESI^+^, TOF, *m*/*z*): [M + H]^+^ calcd for C_32_H_36_O_2_N_2_, 481.2850; found, 481.2875.

**16-(2-Methylphenyl)-3-oxa-9,23-diazaheptacyclo[17.7.1.1^{5,9}^.0^{2,17}^.0^{4,15}^.0^{23,27}^.0^{13,28}^]octacosa-1(27),2(17),4,9(28),13,15,18-heptaen-9-ium (HR101, 10):** 9,9'-[(2-Methylphenyl)methylene]bis(2,3,6,7-tetrahydro-1*H*,5*H*-pyrido[3,2,1-*ij*]quinolin-8-ol) (**15**, 91 mg, 0.19 mmol) and chloranil (69 mg, 0.28 mmol) in MeOH/CHCl_3_ (1:1, 10 mL) were stirred at 23 °C for 1 h. When the reaction had been complete as evidenced by TLC, the solvent was removed under reduced pressure, and the residue was applied directly to a column of silica gel. Flash chromatography (MeOH/CHCl_3_, 1:9) afforded **10** (27 mg, 29%) as a purple film of the chloride salt. ^1^H NMR (400 MHz, CDCl_3_) δ 7.44–7.25 (m, 3H), 7.07 (d, *J =* 6.9 Hz, 1H), 6.56 (s, 2H), 3.41 (m, 8H), 2.97 (t, *J =* 6.2 Hz, 4H), 2.58 (t, *J =* 5.9 Hz, 4H), 2.01 (p, *J =* 6.3 Hz, 4H), 1.90 (s, 3H), 1.84 (p, *J =* 5.3 Hz, 4H); ^13^C NMR (75 MHz, CDCl_3_) δ 156.0, 153.6, 152.6, 137.2, 133.9, 131.6, 130.2, 127.1 (× 2), 125.5, 113.9, 106.7, 51.8, 51.4, 28.5, 21.7, 20.9, 20.8, 19.6; IR (film) ν_max_: 2923, 1682, 1596, 1298 cm^−1^; HRMS (ESI^+^, TOF, *m*/*z*): [M]^+^ calcd for C_32_H_33_ON_2_, 461.2587; found, 461.2580.

### Determination of log *D* values

Values of log *D* were measured by dissolution of solids in 1:1 octanol/phosphate buffer (4 mL total volume, 5 mM phosphate, pH 7.4) to provide 5 mM solutions of the fluorophore. Samples were shaken for 24 h at 25 °C to equilibrate. The top and bottom 1 mL fractions were isolated and centrifuged at 15,000 rpm (no precipitate was observed for any of the samples). Aliquots of these fractions were diluted in octanol (derived from the top fraction) or buffer (derived bottom fraction) to generate stock solutions (10×) appropriate for analysis. These stock solutions were further diluted 10-fold and analyzed in a solvent mixture comprising ethanol (80%), octanol (10%), and phosphate buffer (10%, pH 7.4). The ratio of fluorescence emission at λ_max_ was used to calculate log *D*.

### Biological evaluation

The N2 strain of *C. elegans* was cultured as described [47] and maintained at 20–22.5 °C. Fluorophores were added to normal growth media (NMG) liquid (at 45–55 °C), poured into Petri dishes (60 mm), and allowed to cool until solidified. Fluorophores, were prepared fresh as DMSO stocks (1000×) from dry powders and used immediately. The final amount of DMSO exposed to animals did not exceed 0.1%. Storage of fluorophores as frozen solutions in DMSO is not recommended and can result in loss of potency. Prepared media were seeded with the normal food source of OP50 *E. coli* and incubated overnight at room temperature to dry. Adult *C. elegans* animals (10–20) were added to the media and allowed to feed freely for 2 h prior to imaging. Animals were transferred and imaged on 10% agarose pads in the presence of 2.5% (w/v) polystyrene beads (50 nm, Bangs Laboratory) to prevent movement. Imaging employed an Olympus FV1000 laser scanning confocal microscope and Fluoview software. Images were acquired with Plan-apochromat objectives.

## Supporting Information

File 1 Supplementary time-lapse confocal microscopy video showing dynamics of mitochondrial fusion and fission in a living adult *C. elegans* animal imaged after treatment with HRB (**9**, 1 nM) for 2 h followed by immobilization on an agarose pad containing polystyrene beads. Frames were acquired every 3.3 seconds, and are animated at 3 frames per second. The animal in the video is oriented such that the ventral side is left and the anterior is toward the top of the frame. The distal (mitotic) gonad arm is on the right half of the animal, whereas the proximal oocytes are on the left. Dynamic motility of mitochondria in the gonad including elongation, collapse, fusion, and fission can be observed.
